# Association of hemoglobin, albumin, lymphocyte, and platelet score with risk of all-cause and cause-specific mortality among cancer survivors: NHANES 1999-2018

**DOI:** 10.3389/fonc.2024.1402217

**Published:** 2024-09-18

**Authors:** Jixin Fu, Xiaohan Yue, Yanan Zou, Jian Zhang, Xinjian Wang, Dianliang Zhang

**Affiliations:** ^1^ Department of Gastrointestinal Surgery, Weihai Central Hospital, Qingdao University, Weihai, Shandong, China; ^2^ Department of Pediatric Surgery, Weihai Central Hospital, Qingdao University, Weihai, Shandong, China; ^3^ Department of Anesthesiology, Weihai Central Hospital, Qingdao University, Weihai, Shandong, China; ^4^ Department of Gastrointestinal Surgery, the Affiliated Hospital of Qingdao University, Pingdu, Shandong, China; ^5^ Center of Colon and Rectum, Qingdao Municipal Hospital, Qingdao University, Qingdao, Shandong, China

**Keywords:** cancer survivors, HALP score, all-cause mortality, cause-specific mortality, national health and nutrition examination survey

## Abstract

**Background:**

The HALP score, comprising hemoglobin, albumin, lymphocyte, and platelet levels, serves as an indicator of both nutritional and inflammatory status. However, its correlation with all-cause and cause-specific mortality among cancer survivors remains unclear. Therefore, this study aims to investigate the relationship between HALP scores and mortality outcomes in this population.

**Method:**

We extracted cohort data spanning ten cycles (1999-2018) from the U.S. National Health and Nutrition Examination Survey (NHANES). Mortality rates, determined using the National Death Index (NDI) as of December 31, 2019, were assessed. Weighted multivariate logistic regression analyzed the association between HALP scores and cancer prevalence. Kaplan-Meier analyses and weighted multivariate-adjusted Cox analyses investigated the link between HALP scores and all-cause and cause-specific mortality in cancer survivors. Restricted cubic spline (RCS) analysis was employed to assess nonlinear relationships. Furthermore, multi-parametric subgroup analyses were conducted to ensure the robustness of the results.

**Results:**

Our study included 41,231 participants, of whom 3,786 were cancer survivors (prevalence: 9.5%). Over a median follow-up of 91 months (range: 51-136), we observed 1,339 deaths, including 397 from cancer, 368 from cardio-cerebrovascular disease, and 105 from respiratory disease. Elevated HALP scores showed a consistent association with reduced cancer incidence (P for trend <0.001). In multivariable-adjusted Cox regression analyses, HALP scores were significantly inversely associated with all-cause mortality, cancer mortality, cardio-cerebrovascular disease mortality, and respiratory disease mortality in cancer survivors (P for trend < 0.05). Nonlinear relationships between HALP scores and all-cause and cause-specific mortality in cancer survivors were evident through RCS regression modeling (P for nonlinearity < 0.01). Kaplan-Meier analyses demonstrated that higher HALP scores were indicative of a poorer prognosis.

**Conclusion:**

Our findings indicate a notable inverse correlation between HALP scores and both all-cause and cause-specific mortality among cancer survivors.

## 1 Introduction

Cancer remains the primary cause of mortality worldwide, particularly prevalent in low- and middle-income nations (1). According to the World Health Organization’s (WHO) 2019 statistics, cancer ranks as the primary or secondary cause of death before age 70 in 112 out of 183 countries, and as the third or fourth leading cause in 23 countries (2). Despite a decline in cancer mortality rates from 1991 to 2021, credited to reduced tobacco use, enhanced cancer detection, and advancements in therapies, the global cancer burden is projected to escalate due to demographic shifts and changes in risk factors such as obesity, sedentary lifestyles, and altered fertility rates (3–8). The increasing number of cancer survivors underscores the pressing need for a robust predictive indicator to monitor and enhance their long-term health outcomes.

The Hemoglobin, Albumin, Lymphocyte, and Platelet (HALP) score, introduced by Chen et al. in 2015, initially predicted prognosis in gastric cancer patients (9). Its potential as a biomarker for various illnesses has since garnered attention. Mounting evidence links nutrition, inflammation, and cancer outcomes (10–13). Matsushita et al. suggested a potential link between diet, nutrition, and prostate cancer, possibly mediated by the gut microbiota (14). Dietary-induced inflammation has been implicated in various cancers, such as CRC, liver cancer, prostate cancer, and kidney cancer (15–17). Recent research underscores the significant impact of diet on both mucosal and systemic immune systems, influencing inflammation in tumor cells and their response to cancer therapy (18).

Given HALP score’s ability to assess immune and nutritional status, it can reflect tumor tolerance by integrating immune markers, nutrition, and inflammation. While studies have explored its predictive significance in different cancers, findings vary (19–21). In a multicenter study, lower HALP values correlated with increased risks of mortality and cancer-related deaths in patients with locally advanced colorectal cancer (19). However, a separate study found no significant association between HALP scores and long-term survival in patients with retroperitoneal soft tissue sarcoma (21). Therefore, a comprehensive investigation into HALP score’s association with all-cause and cause-specific mortality in cancer survivors is crucial.

Drawing on NHANES data spanning 1999 to 2018, our cohort study delves into the correlation between the HALP score and all-cause and cause-specific mortality in cancer survivors and to assess the impact of the HALP score on cancer survivors. Our study will provide valuable reference metrics for optimizing treatment and clinical management of cancer survivors.

## 2 Methods

### 2.1 Study population

The NHANES, conducted by the National Center for Health Statistics (NCHS), is a nationally representative cross-sectional study employing a stratified multistage random sample design to assess the health and nutritional status of the US population (22). Before implementation, NHANES surveys undergo thorough review and approval by the Disclosure Review Board of the NCHS. Comprehensive details regarding ethical approval and informed consent procedures are available through the NCHS (23). This study employed a nationwide cross-sectional design, utilizing secondary analyses of publicly accessible and deidentified NHANES data. As such, additional institutional review board approval or informed consent was not required. Additional details can be found at http://www.cdc.gov/nchs/nhanes.

**Figure 1:**
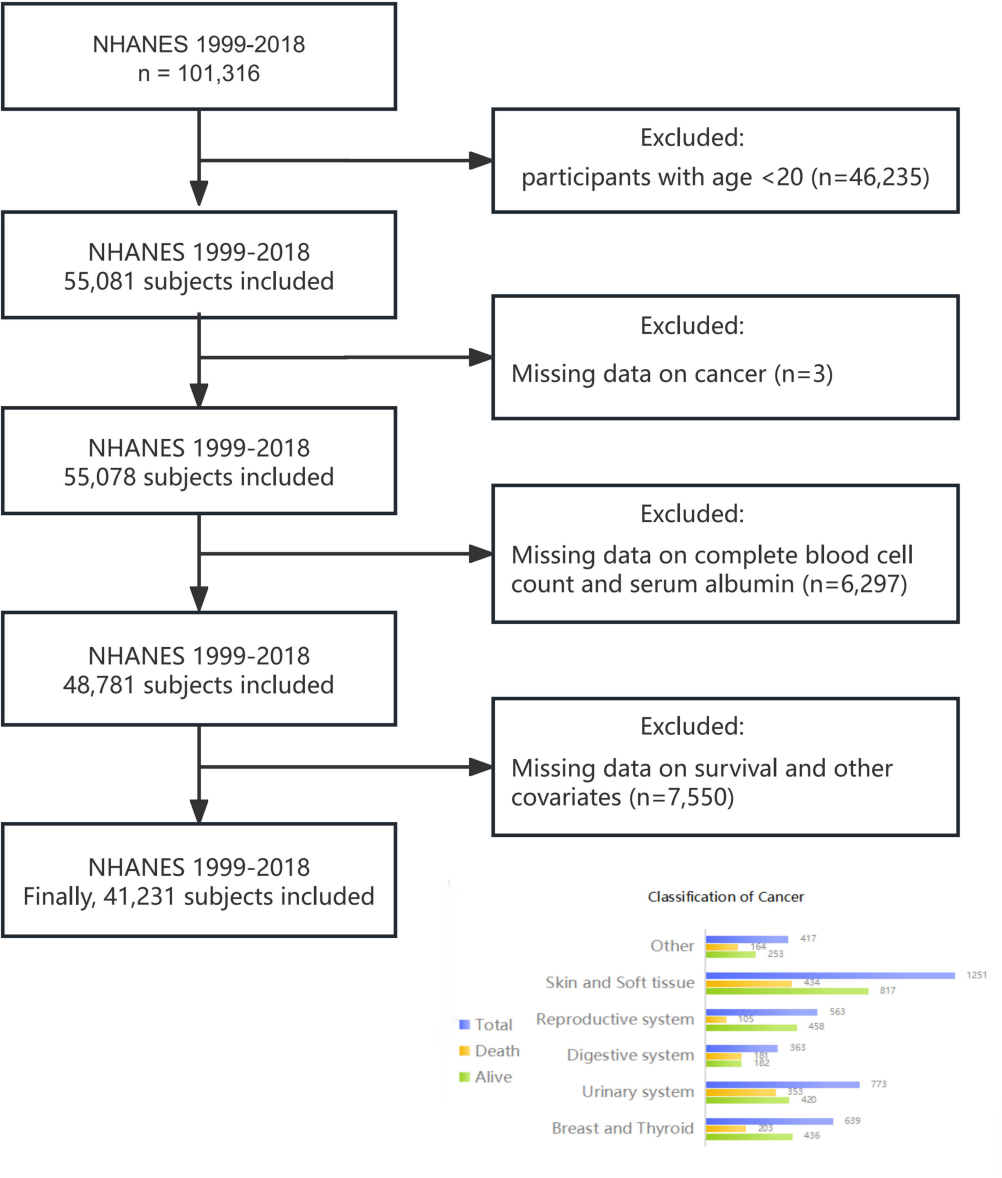
Flowchart of study design.

For our cohort study, we included 101,316 participants from ten NHANES cycles spanning 1999 to 2018. Exclusion criteria encompassed individuals under 20 years old, pregnant individuals, those with missing cancer or HALP score data, and participants with incomplete follow-up or covariate information. Ultimately, 41,231 participants were included in the study (**Figure 1**).

### 2.2 Definition of hemoglobin, albumin, lymphocyte, platelet score

Blood samples were collected during examinations at Mobile Examination Centers (MECs) and subsequently analyzed in the laboratory. The HALP score comprises serum levels of hemoglobin, albumin, lymphocytes, and platelets. Hemoglobin, lymphocyte, and platelet levels are measured using a hematology-analyzing device (UniCel DxH 800 analyzer), while serum albumin levels are determined using Roche Modular P and Roche Cobas 6000 chemistry analyzers. The HALP score is calculated using the formula: hemoglobin (g/L) × albumin (g/L) × lymphocytes (10^9^/L)/platelets (10^9^/L) (24, 25).

### 2.3 Definition of cancer survivor

Self-reported cancer history data were sourced from the “Medical Conditions” section of NHANES, gathered via professionally self-administered questionnaires (26). Cancer survivors were identified by their response to the question: “Have you ever been told by a doctor or other health professional that you had cancer or malignancy of any kind?” Positive responses categorized individuals as cancer survivors, while negative responses classified them as non-cancer individuals. Cancer survivors were categorized based on their responses to the question “What kind of cancer?” The classification comprised six categories, as illustrated in **Figure 1**.

### 2.4 Mortality outcomes

Mortality data were obtained from NHANES Public-Use Linked Mortality Files, available until December 31, 2019. Causes of death were documented using ICD-10 (International Statistical Classification of Diseases, 10th version) codes (27). Our analysis focused on all-cause and cause-specific deaths, including malignant neoplasms (ICD-10: C00-C97), cardio−cerebrovascular disease (ICD-10: I00-I09, I11, I13, I20-I51, I60-I69), and respiratory diseases (ICD-10: J40-J47, J09-J18). The follow-up period for the study extended from the date of initial diagnosis until the date of death or December 31, 2019, whichever came first.

### 2.5 Definitions of covariates

Covariates in this cohort study included age (years), gender (male or female), ethnicity (Mexican American, other Hispanic, non-Hispanic white, non-Hispanic black, or other race), educational attainment (below high school, high school, or above high school), marital status (married/cohabiting, widowed/divorced/separated, or never married), and body mass index (BMI) categories (<18.5, 18.5-25.0, 25.0-29.9, or >29.9 kg/m²) (28). Income was assessed using the Poverty Income Ratio (PIR), classified as ≤1.0, 1.1-3.0, and >3.0 based on US Department of Health and Human Services guidelines. Never smokers were individuals who had smoked fewer than 100 cigarettes in their lifetime. Those who had smoked more than 100 cigarettes and were currently smoking were classified as current smokers, while those who had smoked more than 100 cigarettes but had quit were classified as former smokers (29). Alcohol consumption was dichotomized into non-drinker or drinker (≥12 drinks in a year). Physical activity was quantified as metabolic equivalent (MET) minutes of moderate to vigorous exercise per week according to World Health Organization guidelines (30). Diabetes mellitus was determined by self-report, glycated hemoglobin ≥6.5%, or fasting blood glucose ≥126 mg/dL (7.0 mmol/L). Hypertension was defined by medication use or self-reported diagnosis. Complete blood count parameters, serum albumin levels, high-density lipoprotein cholesterol (HDL-C), and total cholesterol (TG) levels were also collected from the database.

### 2.6 Statistical analysis

We utilized NHANES-recommended weights, specifically the 2-year cycle of Mobile Examination Center (MEC) exam weights (wtmec2yr), for statistical analyses, given that HALP scores were derived from four laboratory measurements. Continuous data were presented as medians [first quantile (P25) and third quantile (P75)] and compared using the nonparametric Wilcoxon rank sum test or independent samples t-test, as applicable. Categorical variables were reported as percentages (%) and assessed using the chi-squared test or Fisher’s exact test, if appropriate.

In this cohort analysis, three logistic regression models were employed to estimate adjusted odds ratios (ORs) and 95% confidence intervals (CIs) for the association between HALP scores and cancer prevalence. Similarly, three Cox regression models were utilized to calculate adjusted hazard ratios (HRs) and 95% CIs for all-cause mortality, cancer mortality, cardio-cerebrovascular disease mortality, and respiratory disease mortality among cancer survivors. Model 1 was unadjusted, while Model 2 was adjusted for age, sex, and race/ethnicity. Model 3 included additional adjustments for education level, family poverty income ratio, MET minutes per week, drinking status, smoking status, BMI, self-reported diabetes, and self-reported hypertension.

Restricted cubic spline regression analyses were conducted to explore dose-response relationships between HALP scores and all-cause and cause-specific mortality among cancer survivors, with knots placed at the 5th, 35th, 65th, and 95th percentiles of each exposure variable. Kaplan-Meier analyses were utilized to evaluate the association between HALP scores and long-term mortality in cancer survivors. Additionally, subgroup analyses were performed to investigate the relationship between HALP scores and mortality outcomes based on age, sex, BMI, smoking status, self-reported hypertension, self-reported diabetes, and different types of cancer.

## 3 Results

### 3.1 Baseline characteristics

**Table 1** presents baseline characteristics and weighted estimates of the study population. Our analysis included 41,231 individuals, representing 17.04 million noninstitutionalized US residents. Among them, 3,786 were cancer survivors, aged 20 to 85 years, with a mean age of 49.47 ± 18.14 years. Race distribution was as follows: Mexican Americans: 2.2%, Other Hispanics: 2.3%, Non-Hispanic Whites people: 87%, Non-Hispanic Blacks people: 5.0%, and Others: 3.4%. Cancer survivors were more likely to be older women, highly educated, higher-income earners, divorced, smokers, and drinkers. Additionally, they exhibited higher waist circumferences, lower physical activity levels, and a higher prevalence of comorbid hypertension or diabetes. Median (P25, P75) values of hemoglobin, serum albumin, lymphocyte count, platelet count, and HALP scores in cancer survivors were 14.10 (13.20, 14.90) g/dL, 4.20 (4.00, 4.40) g/dL, 1.80 (1.50, 2.40)×10^9^/L, 235 (198, 280)×10^9^/L, and 46 (35, 62), respectively, significantly lower than non-cancer participants. Significant differences in HALP scores and HALP-related parameters were observed between individuals with and without cancer (P< 0.05).

**Table 1:** Baseline characteristics of adults in NHANES 1999–2018.

Characteristics	Overall, N = 41231 (100%)	Cancer survivors		P Value
		No, N = 37445 (91%)	Yes, N = 3786 (9.5%)	
Sex, %				<0.001
Female	21,245 (52%)	19,251 (51%)	1,994 (58%)	
Male	19,986 (48%)	18,194 (49%)	1,792 (42%)	
Age, %				<0.001
20-35 years	10,733 (28%)	10,562 (30%)	171 (5.0%)	
35-60 years	16,754 (48%)	15,858 (49%)	896 (34%)	
60+ years	13,744 (25%)	11,025 (21%)	2,719 (61%)	
Race/ethnicity, %				<0.001
Non-Hispanic White	18,748 (69%)	16,036 (67%)	2,712 (87%)	
Non-Hispanic Black	8,269 (11%)	7,782 (11%)	487 (5.0%)	
Mexican American	7,082 (8.3%)	6,834 (8.9%)	248 (2.2%)	
Other Race - Including Multi-Racial	3,686 (6.9%)	3,535 (7.2%)	151 (3.4%)	
Other Hispanic	3,446 (5.3%)	3,258 (5.6%)	188 (2.3%)	
Education level, %				<0.001
Below high school	10,876 (17%)	10,019 (17%)	857 (14%)	
High school	9,580 (24%)	8,711 (24%)	869 (22%)	
Above high school	20,775 (59%)	18,715 (59%)	2,060 (64%)	
Marital status, %				<0.001
Married/cohabiting	25,000 (64%)	22,680 (64%)	2,320 (66%)	
Widowed/divorced/separated	9,066 (18%)	7,823 (17%)	1,243 (28%)	
Never married	7,165 (17%)	6,942 (19%)	223 (5.6%)	
Family PIR, %				<0.001
≤1.0	7,790 (13%)	7,307 (14%)	483 (8.6%)	
1.1–3.0	19,301 (40%)	17,493 (40%)	1,808 (39%)	
>3.0	14,140 (47%)	12,645 (46%)	1,495 (52%)	
Smoking status, %				<0.001
Never smoker	22,345 (54%)	20,669 (55%)	1,676 (45%)	
Former smoker	10,200 (25%)	8,682 (23%)	1,518 (38%)	
Current smoker	8,685 (21%)	8,093 (22%)	592 (17%)	
Drinking status, %				<0.001
Drinker	5,332 (11%)	4,743 (11%)	589 (14%)	
Nondrinker	35,899 (89%)	32,702 (89%)	3,197 (86%)	
Body mass index, %				0.3
Underweight, kg/m^2^	647 (1.6%)	585 (1.6%)	62 (1.7%)	
Normal, kg/m^2^	11,339 (29%)	10,335 (30%)	1,004 (28%)	
Obese, kg/m^2^	13,750 (34%)	12,440 (33%)	1,310 (35%)	
Overweight, kg/m^2^	14,761 (35%)	13,454 (35%)	1,307 (35%)	
Age, years	46.0 (33.0, 59.0)	44.0 (32.0, 57.0)	64.0 (53.0, 75.0)	<0.001
Family PIR	2.98 (1.49, 5.00)	2.93 (1.46, 5.00)	3.40 (1.81, 5.00)	<0.001
BMI , kg/m2	28 (24, 32)	28 (24, 32)	28 (24, 32)	0.3
Waist Circumference (cm)	97 (87, 108)	97 (86, 108)	100 (90, 111)	<0.001
Self-reported hypertension, %	14,286 (31%)	12,162 (29%)	2,124 (51%)	<0.001
Self-reported diabetes, %	6,124 (11%)	5,305 (10%)	819 (17%)	<0.001
MET minute/week	1,890 (0, 5,460)	2,100 (0, 5,460)	1,680 (0, 5,040)	<0.001
Hemoglobin (g/dL)	14.30 (13.30, 15.40)	14.40 (13.40, 15.40)	14.10 (13.20, 14.90)	<0.001
Albumin (g/dL)	4.30 (4.10, 4.50)	4.30 (4.10, 4.50)	4.20 (4.00, 4.40)	<0.001
Platelet count (10^9^/L)	246 (209, 290)	247 (210, 291)	235 (198, 280)	<0.001
Lymphocyte count (10^9^/L)	2.00 (1.60, 2.50)	2.00 (1.70, 2.50)	1.80 (1.50, 2.40)	<0.001
HDL-C, mg/dl	51 (41, 63)	51 (41, 62)	52 (42, 65)	0.003
Total cholesterol, mg/dl	194 (168, 222)	194 (168, 222)	196 (170, 225)	0.2
HALP score	50 (39, 65)	51 (39, 66)	46 (35, 62)	<0.001
HALP score classifcation				<0.001
Tertile 1	13,606 (31%)	12,012 (30%)	1,594 (40%)	
Tertile 2	13,606 (34%)	12,470 (35%)	1,136 (31%)	
Tertile 3	14,019 (35%)	12,963 (35%)	1,056 (29%)	

PIR, poverty income ratio; HDL-C, High-density lipoprotein cholesterol; HALP score, Hemoglobin, albumin, lymphocyte, and platelet score.

Continuous variables are described as medians [interquartile ranges]. Categorical variables are presented as numbers (percentages). N reflect the study sample while percentages reflect the survey-weighted.

### 3.2 HALP score and cancer prevalence

**Table 2** illustrates the relationship between HALP scores and cancer prevalence using weighted multivariate regression models. All three logistic regression models revealed a negative association between HALP score and cancer incidence. The ORs and 95% CIs for the highest tertile compared to the lowest tertile were as follows: OR=0.61 (0.57-0.67), p for trend<0.001; OR=0.83 (0.75-0.89), p for trend<0.001; and OR=0.61 (0.75-0.89), p for trend<0.001, respectively.

**Table 2:** Logistic regression analysis between HALP score and prevalence of cancer among adults in NHANES 1999–2018.

	HALP score			P for trend
	Tertile 1	Tertile 2	Tertile 3	
Range	<41.41	41.41-58.47	>58.47	
Crude	1.00 [Reference]	0.69 (0.63, 0.74)	0.61 (0.57, 0.67)	<0.001
Model 1	1.00 [Reference]	0.81 (0.74, 0.88)	0.81 (0.75, 0.89)	<0.001
Model 2	1.00 [Reference]	0.81 (0.74, 0.88)	0.83 (0.75, 0.89)	<0.001

Data are presented as OR (95% CI); Model 1 was adjusted as age (continuous), MET (continuous), sex (male or female), and race/ethnicity (Mexican American, Other Hispanic, Non-Hispanic White, Non-Hispanic Black or Other); Model 2 was adjusted as model 1 plus education level (below high school, high school, or above high school), family poverty income ratio (≤1.0,1.1–3.0, or >3.0), drinking status (nondrinker, drinker), smoking status (never smoker, former smoker, or current smoker), BMI (<18.5, 18.5- 25.0, 25.0-29.9, or >29.9), self-reported diabetes (yes or no), and self-reported hypertension (yes or no).

### 3.3 HALP score and mortality

Over a median follow-up of 91 (51, 136) months, 1,339 (35.37%) out of 3,786 cancer survivors succumbed to all-cause mortality, with 397 (10.49%) attributed to cancer, 367 (9.70%) to cardio-cerebrovascular disease, and 105 (2.77%) to respiratory disease. As shown in **Table 3**, higher HALP scores were significantly associated with a reduced risk of all-cause mortality and cause-specific mortality among survivors, evident in both crude and multivariable-adjusted Models 1 and 2 (all p for trend <0.05). The multivariable-adjusted HRs and 95% CIs for the highest tertile compared with the lowest tertile for all-cause mortality, cancer mortality, cardio-cerebrovascular disease mortality, and respiratory disease mortality among cancer survivors were 0.61 (0.49-0.76), 0.91 (0.70-1.17), 0.67 (0.49-0.91), and 0.60 (0.35-0.97), respectively.

**Table 3:** Cox regression analysis between HALP score and long-term mortality among cancer survivor in NHANES 1999–2018.

	HALP score			P for trend
	Tertile 1	Tertile 2	Tertile 3	
All-cause mortality				
No. deaths/total	670/1594	357/1136	312/1056	
Crude	1.00 [Reference]	0.63 (0.54, 0.74)	0.58 (0.49, 0.68)	<0.001
Model 1	1.00 [Reference]	0.66 (0.54, 0.80)	0.68 (0.55, 0.83)	<0.001
Model 2	1.00 [Reference]	0.64 (0.51, 0.79)	0.61 (0.49, 0.76)	<0.001
Cancer mortality				
No. deaths/total	190/1594	97/1136	110/1056	
Crude	1.00 [Reference]	0.69 (0.53, 0.89)	0.86 (0.67, 1.10)	0.016
Model 1	1.00 [Reference]	0.72 (0.56, 0.94)	0.95 (0.73, 1.22)	0.040
Model 2	1.00 [Reference]	0.72 (0.55, 0.93)	0.91 (0.70,1.17)	0.038
Cardio−cerebrovascular disease mortality				
No. deaths/total	196/1368	94/1367	77/1367	
Crude	1.00 [Reference]	0.64 (0.50, 0.83)	0.56 (0.42, 0.74)	<0.001
Model 1	1.00 [Reference]	0.72 (0.54, 0.95)	0.70 (0.52, 0.95)	0.019
Model 2	1.00 [Reference]	0.70 (0.52, 0.92)	0.67 (0.49, 0.91)	0.008
Respiratory disease mortality				
No. deaths/total	62/1368	20/1367	23/1367	
Crude	1.00 [Reference]	0.44 (0.26, 0.72)	0.55 (0.33, 0.88)	0.002
Model 1	1.00 [Reference]	0.50 (0.30, 0.80)	0.67 (0.41, 1.07)	0.011
Model 2	1.00 [Reference]	0.47 (0.27, 0.78)	0.60 (0.35, 0.97)	0.006

Data are presented as HR (95% CI); Model 1 was adjusted as age (continuous), MET (continuous), sex (male or female), and race/ethnicity (Mexican American, Other Hispanic, Non-Hispanic White, Non-Hispanic Black or Other); Model 2 was adjusted as model 1 plus education level (below high school, high school, or above high school), family poverty income ratio (≤1.0,1.1–3.0, or >3.0), drinking status (nondrinker, drinker), smoking status (never smoker, former smoker, or current smoker), BMI (<18.5, 18.5- 25.0, 25.0-29.9, or >29.9), self-reported diabetes (yes or no), and self-reported hypertension (yes or no).

**Figure 2:**
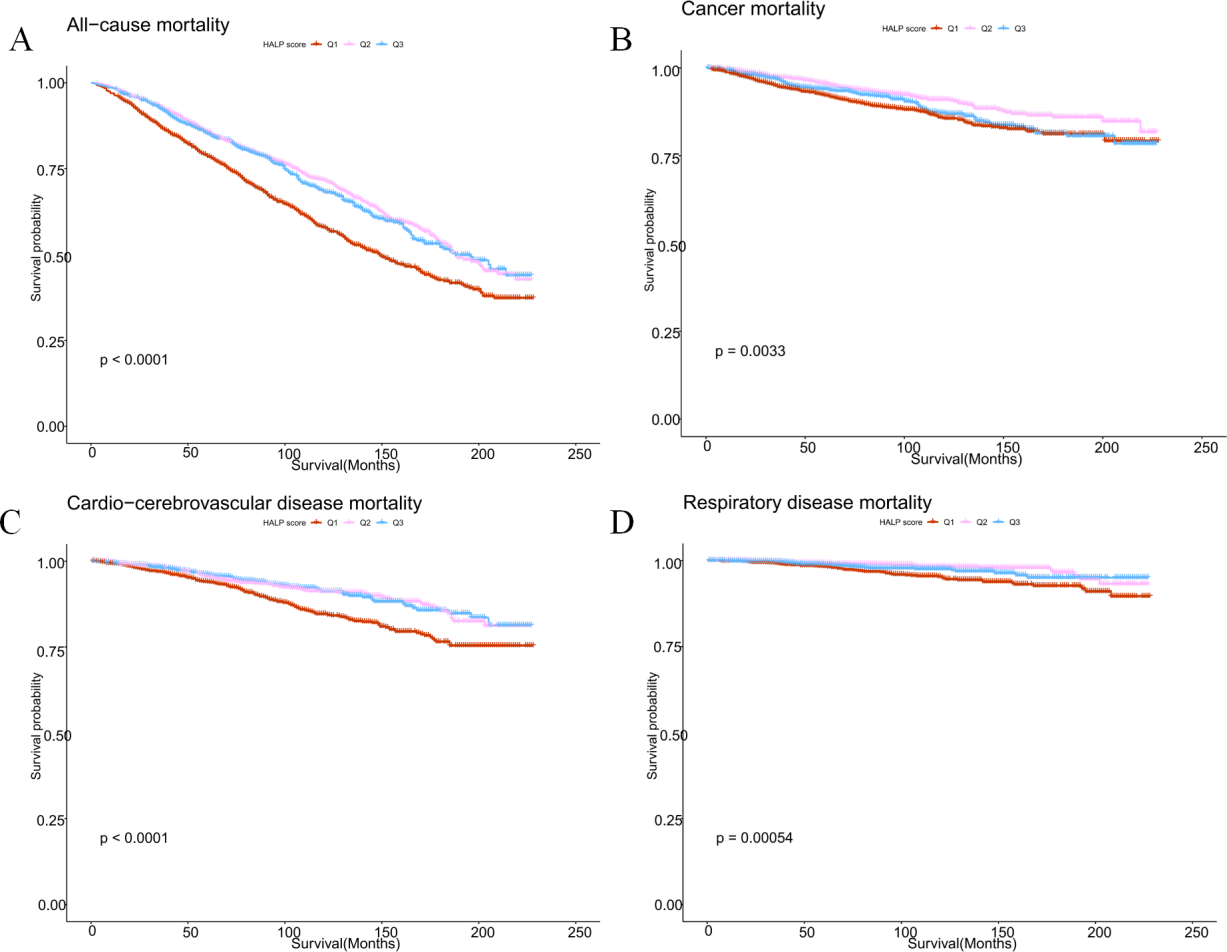
Kaplan-Meier survival survival estimates between HALP scores and all-cause mortality **(A)**, cancer mortality **(B)**, cardio-cerebrovascular disease mortality **(C)**, and respiratory disease mortality **(D)** in cancer survivors.

Furthermore, Kaplan-Meier analysis revealed a comprehensive association between HALP scores and both cause-specific and all-cause mortality in cancer survivors. Higher HALP scores were correlated with reduced all-cause mortality (p=0.0033), decreased cancer mortality (p<0.0001), lowered cardio-cerebrovascular disease mortality (p<0.0001), and lessened respiratory disease mortality (p=0.00054) among cancer survivors (**Figure 2**).

### 3.4 Restricted cubic spline analysis

**Figure 3:**
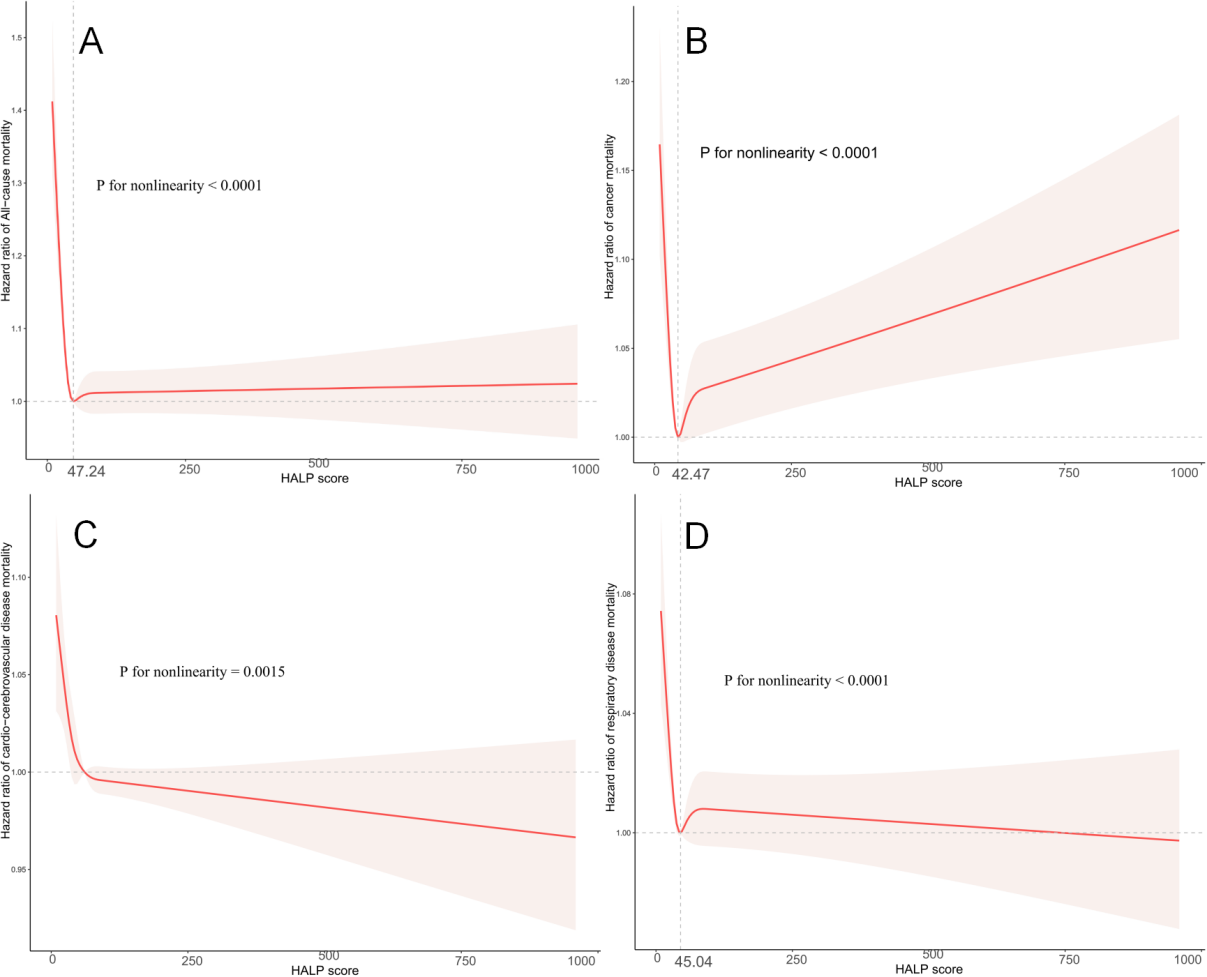
Restricted cubic spline analysis to assess the association between HALP score and all-cause mortality **(A)**, cancer mortality **(B)**, cardio-cerebrovascular disease mortality **(C)**, and respiratory disease mortality **(D)** in cancer survivors. Adjusted for age (continuous), MET (continuous), sex (male or female), ethnicity (Mexican American, Other Hispanic, Non-Hispanic White, Non-Hispanic Black or Other), education level (below high school, high school, or above high school), family poverty income ratio (≤1.0, 1.1–3.0, or >3.0), drinking status (nondrinker, drinker), smoking status (never smoker, former smoker, or current smoker), BMI (<18.5, 18.5- 25.0, 25.0-29.9, or >29.9), self-reported diabetes (yes or no), and self-reported hypertension (yes or no).

We employed weighted restricted cubic spline curves to explore the nonlinear relationship between HALP scores and all-cause, as well as cause-specific mortality, while controlling for potential confounders. Illustrated in **Figure 3**, the restricted cubic spline analysis unveiled a nonlinear association between HALP scores and cardio-cerebrovascular disease mortality in cancer survivors (nonlinear P=0.0015). Notably, lower HALP scores were linked to an elevated risk of cardio-cerebrovascular disease mortality in this population.

Furthermore, nonlinear associations were also observed between HALP scores and all-cause mortality (nonlinear P<0.0001), cancer mortality (nonlinear P<0.0001), and respiratory disease mortality (nonlinear P<0.0001) in cancer survivors. Importantly, the results of the two linear regressions indicated that the probability of all-cause mortality, cancer mortality, and respiratory disease mortality progressively decreased to the lowest point at HALP scores of 47.24, 42.47, and 45.04, respectively, before increasing with rising HALP scores.

### 3.5 Subgroup analyses

To further evaluate the robustness of the relationship between HALP scores and all-cause, as well as cause-specific mortality in cancer survivors, subgroup analyses were conducted based on sex, age, BMI, smoking, hypertension status, and diabetes status. The findings indicated a largely consistent dose-response relationship between HALP scores and all-cause and cause-specific mortality across subgroups, particularly in all-cause mortality. However, the association between HALP scores and cancer mortality in the subgroup analyses was less pronounced (**Table 4**). Moreover, no statistically significant interaction p-values were detected (**Table 4**).

**Table 4:** Subgroup analysis of HALP score and long-term mortality among cancer survivor in NHANES 1999–2018.

	HALP score			P for trend	P for interaction
	Tertile 1	Tertile 2	Tertile 3		
All-cause mortality					
Age					0.835
<60 years	1.00 [Reference]	0.55 (0.31, 0.95)	0.52 (0.30, 0.87)	0.025	
>60 years	1.00 [Reference]	0.62 (0.51, 0.75)	0.58 (0.47, 0.71)	<0.001	
Sex					0.719
Male	1.00 [Reference]	0.59 (0.45, 0.76)	0.56 (0.43, 0.74)	<0.001	
Female	1.00 [Reference]	0.69 (0.46, 1.03)	0.64 (0.41, 0.99)	0.037	
BMI					0.364
<25.0	1.00 [Reference]	0.56 (0.37, 0.85)	0.85 (0.55, 1.31)	0.024	
25.0-29.9	1.00 [Reference]	0.71 (0.51, 0.98)	0.75 (0.53, 1.06)	0.079	
>29.9	1.00 [Reference]	0.74 (0.50, 1.09)	0.55 (0.36, 0.82)	0.015	
Smoke					0.586
Yes	1.00 [Reference]	0.68 (0.53, 0.87)	0.61 (0.47, 0.78)	<0.001	
No	1.00 [Reference]	0.58 (0.38, 0.87)	0.67 (0.43, 1.03)	0.021	
Diabetes					0.389
Yes	1.00 [Reference]	0.75 (0.51, 1.09)	0.64 (0.43, 0.95)	0.037	
No	1.00 [Reference]	0.61 (0.47, 0.79)	0.64 (0.49, 0.84)	<0.001	
Hypertension					0.158
Yes	1.00 [Reference]	0.73 (0.56, 0.96)	0.62 (0.46, 0.82)	0.002	
No	1.00 [Reference]	0.55 (0.39, 0.76)	0.70 (0.49, 0.98)	0.001	
Cancer mortality					
Age					0.711
<60 years	1.00 [Reference]	0.80 (0.36, 1.69)	0.76 (0.36, 1.60)	0.700	
>60 years	1.00 [Reference]	0.68 (0.51, 0.91)	0.90 (0.68, 1.18)	0.028	
Sex					0.640
Male	1.00 [Reference]	0.69 (0.49, 0.96)	0.81 (0.59, 1.13)	0.041	
Female	1.00 [Reference]	0.76 (0.50, 1.16)	1.03 (0.67, 1.57)	0.400	
BMI					0.306
<25.0	1.00 [Reference]	0.78 (0.47, 1.28)	1.14 (0.69, 1.84)	0.400	
25.0-29.9	1.00 [Reference]	0.87 (0.55, 1.36)	1.19 (.76, 1.86)	0.500	
>29.9	1.00 [Reference]	0.63 (0.39, 0.98)	0.73 (0.46, 1.13)	0.100	
Smoke					0.662
Yes	1.00 [Reference]	0.72 (0.52, 0.98)	0.86 (0.63, 1.17)	0.120	
No	1.00 [Reference]	0.71 (0.44, 1.11)	1.07 (0.68, 1.67)	0.200	
Diabetes					0.263
Yes	1.00 [Reference]	0.86 (0.51, 1.44)	0.82 (0.47, 1.40)	0.700	
No	1.00 [Reference]	0.69 (0.51, 0.93)	1.00 (0.75, 1.34)	0.032	
Hypertension					0.921
Yes	1.00 [Reference]	0.76 (0.54, 1.05)	0.95 (0.68, 1.31)	0.200	
No	1.00 [Reference]	0.71 (0.46, 1.08)	0.96 (0.63, 1.45)	0.200	
Cardio−cerebrovascular disease mortality					
Age					0.711
<60 years	1.00 [Reference]	0.46 (0.17, 1.14)	0.17 (0.04, 0.50)	0.004	
>60 years	1.00 [Reference]	0.67 (0.51, 0.88)	0.65 (0.49, 0.87)	0.002	
Sex					0.640
Male	1.00 [Reference]	0.65 (0.43, 0.97)	0.68 (0.44, 1.02)	0.045	
Female	1.00 [Reference]	0.77 (0.52, 1.11)	0.64 (0.41, 0.98)	0.044	
BMI					0.306
<25.0	1.00 [Reference]	0.66 (0.39, 1.08)	0.85 (0.51, 1.38)	0.300	
25.0-29.9	1.00 [Reference]	0.60 (0.36, 0.99)	0.67 (0.38, 1.15)	0.100	
>29.9	1.00 [Reference]	0.85 (0.53, 1.35)	0.60 (0.36, 1.00)	0.150	
Smoke					0.662
Yes	1.00 [Reference]	0.75 (0.52, 1.07)	0.78 (0.53, 1.12)	0.200	
No	1.00 [Reference]	0.66 (0.43, 0.98)	0.55 (0.34, 0.88)	0.016	
Diabetes					0.263
Yes	1.00 [Reference]	0.61 (0.36, 1.01)	0.64 (0.37, 1.07)	0.094	
No	1.00 [Reference]	0.77 (0.54, 1.09)	0.70 (0.47, 1.02)	0.120	
Hypertension					0.921
Yes	1.00 [Reference]	0.76 (0.55, 1.05)	0.71 (0.50, 1.01)	0.010	
No	1.00 [Reference]	0.60 (0.33, 1.08)	0.63 (0.33, 1.17)	0.200	
Respiratory disease mortality					
Age					0.798
<60 years	1.00 [Reference]	0.22 (0.04, 0.90)	0.29 (0.08, 0.99)	0.035	
>60 years	1.00 [Reference]	0.47 (0.27, 0.79)	0.61 (0.35, 1.02)	0.010	
Sex					0.395
Male	1.00 [Reference]	0.28 (0.12, 0.58)	0.39 (0.19, 0.77)	<0.001	
Female	1.00 [Reference]	0.67 (0.37, 1.17)	0.78 (0.43, 1.39)	0.300	
BMI					0.313
<25.0	1.00 [Reference]	0.36 (0.14, 0.80)	0.40 (0.16, 0.88)	0.011	
25.0-29.9	1.00 [Reference]	0.62 (0.22, 1.61)	0.71 (0.25, 1.86)	0.600	
>29.9	1.00 [Reference]	0.38 (0.11, 1.04)	0.76 (0.30, 1.80)	0.200	
Smoke					0.981
Yes	1.00 [Reference]	0.49 (0.27, 0.87)	0.60 (0.33, 1.05)	0.030	
No	1.00 [Reference]	0.43 (0.08, 1.55)	0.64 (0.15, 2.14)	0.400	
Diabetes					0.077
Yes	1.00 [Reference]	0.90 (0.28, 2.66)	1.75 (0.65, 4,79)	0.400	
No	1.00 [Reference]	0.39 (0.19, 0.72)	0.40 (0.19, 0.75)	0.001	
Hypertension					0.499
Yes	1.00 [Reference]	0.36 (0.16, 0.75)	0.57 (0.28, 1.10)	0.016	
No	1.00 [Reference]	0.62 (0.31, 1.17)	0.59 (0.28, 1.17)	0.200	

Data are presented as HR (95% CI), which was adjusted as age (continuous), MET (continuous), sex (male or female), race/ethnicity (Mexican American, Other Hispanic, Non-Hispanic White, Non-Hispanic Black or Other), education level (below high school, high school, or above high school), family poverty income ratio (≤1.0,1.1-3.0, or >3.0), drinking status (nondrinker, drinker), smoking status (never smoker, former smoker, or current smoker), BMI (<18.5, 18.5- 25.0, 25.0-29.9, or >29.9), self-reported diabetes (yes or no), and self-reported hypertension (yes or no). In addition, the corresponding subgroup analyses require the exclusion of the corresponding variables (e.g., age subgroup analyses require the exclusion of age).

To delve deeper into the influence of HALP scores across different tumor types, subgroup analyses were conducted according to tumor classification. Results presented in **Table 5** indicate a notable impact of HALP scores on all-cause mortality among patients with thyroid and breast cancers, as well as those with tumors of the digestive system, and skin and soft tissue tumors. This observation suggests a tumor-specific effect of HALP scores on cancer survivors.

**Table 5:** Subgroup analysis by cancer of HALP score and all-cause mortality among cancer survivor in NHANES 1999–2018.

	HALP score			P for trend
	Tertile 1	Tertile 2	Tertile 3	
Breast and Thyroid				
No. deaths/total	118/639	48/639	37/639	
Crude	1.00 [Reference]	0.65 (0.41, 1.01)	0.54 (0.36, 0.80)	0.005
Model 1	1.00 [Reference]	0.76 (0.44, 1.31)	0.53 (0.32, 0.85)	0.031
Model 2	1.00 [Reference]	0.67 (0.37, 1.19)	0.49 (0.29, 0.82)	0.021
Urinary system				
No. deaths/total	174/773	98/773	81/773	
Crude	1.00 [Reference]	0.77 (0.55, 1.08)	0.68 (0.48, 0.97)	0.075
Model 1	1.00 [Reference]	0.76 (0.52, 1.10)	0.93 (0.63, 1.38)	0.3
Model 2	1.00 [Reference]	0.71 (0.48, 1.05)	0.84 (0.56, 1.27)	0.2
Digestive system				
No. deaths/total	103/363	46/363	32/363	
Crude	1.00 [Reference]	0.70 (0.42, 1.14)	0.51 (0.30, 0.87)	0.038
Model 1	1.00 [Reference]	0.59 (0.34, 1.03)	0.47 (0.26, 0.84)	0.023
Model 2	1.00 [Reference]	0.54 (0.29, 1.00)	0.39 (0.20, 0.74)	0.009
Reproductive system				
No. deaths/total	51/563	31/563	23/563	
Crude	1.00 [Reference]	0.73 (0.44, 1.20)	0.52 (0.30, 0.89)	0.052
Model 1	1.00 [Reference]	0.88 (0.48, 1.58)	0.75 (0.39, 1.40)	0.7
Model 2	1.00 [Reference]	0.75 (0.34, 1.60)	0.48 (0.20, 1.13)	0.2
Skin and Soft tissue				
No. deaths/total	190/1251	130/1251	114/1251	
Crude	1.00 [Reference]	0.67 (0.51, 0.89)	0.57 (0.43, 0.76)	<0.001
Model 1	1.00 [Reference]	0.75 (0.61, 1.07)	0.68 (0.56, 0.89)	0.03
Model 2	1.00 [Reference]	0.78 (0.56, 1.08)	0.66 (0.47, 0.93)	0.047
Other				
No. deaths/total	86/417	34/417	44/417	
Crude	1.00 [Reference]	0.53 (0.32, 0.87)	0.56 (0.35, 0.89)	0.011
Model 1	1.00 [Reference]	0.67 (0.37, 1.20)	0.72 (0.41, 1.24)	0.3
Model 2	1.00 [Reference]	0.68 (0.36, 1.26)	0.60 (0.33, 1.07)	0.2

Data are presented as HR (95% CI); Model 1 was adjusted as age (continuous), MET (continuous), sex (male or female), and race/ethnicity (Mexican American, Other Hispanic, Non-Hispanic White, Non-Hispanic Black or Other); Model 2 was adjusted as model 1 plus education level (below high school, high school, or above high school), family poverty income ratio (≤1.0,1.1–3.0, or >3.0), drinking status (nondrinker, drinker), smoking status (never smoker, former smoker, or current smoker), BMI (<18.5, 18.5- 25.0, 25.0-29.9, or >29.9), self-reported diabetes (yes or no), and self-reported hypertension (yes or no).

## 4 Discussion

In our cross-sectional study spanning 1999 to 2018, we investigated the association between HALP scores and cancer prevalence, as well as long-term mortality in the US population. Following adjustment for multiple variables, a significant negative relationship emerged between HALP score and cancer incidence. Among cancer survivors, we observed a notable nonlinear association between HALP score and all-cause, as well as cause-specific mortality (including cancer, cardio-cerebrovascular disease, and respiratory disease mortality), with higher HALP scores correlating with reduced mortality rates. Notably, HALP scores of 47.24, 42.47, and 45.04 corresponded to the lowest all-cause, cancer, and respiratory disease mortality, respectively. Kaplan-Meier analysis further revealed that lower HALP scores were associated with shorter survival times among cancer survivors. Importantly, these findings remained robust across multiple subgroup analyses. In addition, subgroup analyses based on different types of cancer revealed that the effect of HALP scores on all-cause mortality in cancer survivors may be tumor-specific. Overall, our results suggest that HALP score holds significant potential as a valuable predictor of outcomes for cancer survivors.

Immunity and nutrition play pivotal roles in cancer development and progression. Chronic inflammation is known to be carcinogenic, contributing to increased stem cell proliferation and local mutagenic effects through long-term cell turnover stimulation (10, 31, 32). The relationship between neoplasia and the immune system is encapsulated in the ‘3E hypothesis,’ which delineates the stages of initial elimination of transformed cells by immunological effector cells, followed by equilibrium between malignant cells and the immune response within a smoldering neoplastic lesion, and ultimately, the escape of cancer cells from immunological control (33). Systemic inflammation, a hallmark of the tumor microenvironment, significantly influences disease progression and prognosis in cancer survivors (34, 35). Numerous studies have investigated various systemic inflammatory biomarkers and have demonstrated their high predictive value for the prognosis of different cancer types (36–40).

Malnutrition is often linked to tumor progression, stemming from inadequate nutritional intake, increased tumor consumption, or the effects of anticancer therapy. International point prevalence studies report malnutrition rates ranging from 31% to 39% among lower gastrointestinal cancer patients (41, 42). Malnutrition can compromise immunity, trigger metabolic disturbances, and diminish treatment tolerance in cancer survivors, all of which can influence the efficacy of oncological treatment and patient prognosis (43, 44). A study investigating penile cancer patients undergoing inguinal lymph node dissection (ILND) revealed that the preoperative albumin alkaline phosphatase ratio (AAPR) reliably predicts pathologic lymph node-positive (pN+) status (45). Similarly, in bladder cancer patients, an elevated preoperative fibrinogen-to-albumin ratio (FAR) has been identified as a potential predictor of malignancy and advanced grade (46). These findings underscore the significant predictive role of nutritional status in cancer patients.

The significance of inflammatory response and nutritional status in cancer prognosis is increasingly recognized. Nøst et al. (47) analyzed the UK Biobank data and found that the systemic immune-inflammatory index (SII), neutrophil-to-lymphocyte ratio (NLR), and platelet-to-lymphocyte ratio (PLR) correlated positively with the risk of 7 out of 17 cancers, while the lymphocyte-to-monocyte ratio (LMR) correlated negatively. Ouyang et al. (48) identified preoperative SII as an independent prognostic marker in pediatric osteosarcoma. Regarding nutrition, Kheirouri et al. (49) linked high preoperative Controlling Nutritional Status (CONUT) scores to lower overall survival (OS) and cancer-specific survival (CSS) across various cancers. Another study validated the Cholesterol-modified Prognostic Nutritional Index (CPNI) for predicting breast cancer prognosis (50). These findings highlight the combined impact of inflammation and nutrition on cancer outcomes, suggesting that effective prognostic models should integrate both factors.

HALP scores, derived from hemoglobin, albumin, lymphocyte, and platelet levels, serve as indicators of the host’s inflammatory and nutritional status. Hemoglobin, a pivotal factor in tumor progression, is frequently depleted in cancer survivors, contributing to hypoxia (51), a driver of tumor advancement and treatment resistance (52). Numerous studies have established a correlation between hemoglobin levels in cancer patients and both survival outcomes and disease progression (53–55).

Serum albumin, synthesized by the liver, constitutes a crucial component of total serum protein and reflects the host’s inflammatory and nutritional profile. Hypoalbuminemia, stemming from malnutrition, hypermetabolism, systemic inflammation, or heightened cytokine release, compromises the immune response to cancer cells (56). Extensive research has underscored the association between hypoalbuminemia and poor survival across various cancer types (57, 58).

Moreover, lymphocytes play a pivotal role in the host’s anti-cancer defense mechanisms. They secrete cytokines such as interferon-γ and tumor necrosis factor-α (TNF-α), which enhance prognosis by inducing apoptosis and impeding cancer cell proliferation, invasion, and migration (59, 60). Consequently, a decline in lymphocyte levels correlates with a poorer prognosis for cancer survivors.

Additionally, recent research suggests that platelets participate in various signaling pathways implicated in tumor immunity and progression (61). Several studies have demonstrated that elevated pretreatment platelet counts are associated with reduced survival rates in cancer patients (62–64). Taken together, these findings underscore the potential of the HALP score as a valuable prognostic tool for assessing cancer survivors.

The HALP score has demonstrated promising prognostic value across various cancer types, including gastric cancer (9), esophageal squamous cell carcinoma (65), colorectal cancer (19), renal cell carcinoma (66), bladder cancer (20), and small cell lung cancer (67). However, the association between HALP scores and the risk of all-cause and cause-specific mortality across all cancer types remains unexplored. Using NHANES 1999-2018 data, our study unveiled a significant nonlinear relationship between HALP scores and all-cause and cause-specific mortality. Higher HALP scores were consistently associated with reduced all-cause and cause-specific mortality. Notably, HALP scores of 47.24, 42.47, and 45.04 were linked to the lowest levels of all-cause mortality, cancer mortality, and respiratory disease mortality, respectively. Our study bridges this gap in research and provides an invaluable tool for the treatment and management of cancer survivors.

Although survival rates for cancer survivors remain poor, improvements in treatment strategies and care have positively impacted the prognosis of cancer survivors. With increased survival, non-cancer causes of death have become more and more important. In Japan, the reported number of deaths from cancer in 2021 was 381,505 (26.5%) among cancer survivals, and cancer was still the leading cause of death, however, heart disease was the second highest cause of death, accounting for as high as 214,710 (14.91%) recorded deaths, followed by cerebrovascular disease (104,595 deaths, 7.26%) (68). As a result, the proportion of non-cancer mortality among cancer survivors will increase in the future. Thus, a valuable predictive parameter must properly predict not only the risk of all-cause and cancer mortality in cancer survivors but also the risk of non-cancer mortality. To our knowledge, our study represents the first comprehensive investigation into the relationship between HALP scores and all-cause and cause-specific mortality among cancer survivors. Our findings demonstrate significant associations between HALP scores and all-cause mortality, cancer mortality, cardio-cerebrovascular disease mortality, and respiratory disease mortality in this population. Our study introduces a reliable monitoring metric for the management of cancer survivors, facilitating accurate prognosis prediction and enabling timely interventions that can markedly enhance their outcomes.

An interesting observation in our results is that we found a significant effect of the HALP score on all-cause mortality in patients with breast cancer, thyroid cancer, digestive system tumors, and skin and soft-tissue tumors, suggesting that its effect varies across tumor types. Consistent with prior research, Zhao et al. demonstrated the HALP score’s independent prognostic value in early-stage breast cancer, correlating with poorer recurrence-free survival (69). Similarly, Duzkopru et al. identified the HALP score as a prognostic indicator in patients with metastatic gastric cancer (70). These findings underscore the tumor-specific impact of the HALP score on cancer survivors, warranting further investigation into its underlying mechanisms. In addition, in our subgroup analysis, we found a consistently robust correlation between HALP score and all-cause mortality in cancer survivors. However, certain specific mortality results did not reach significance, likely due to the limited data available. Therefore, additional validation through large-scale studies is necessary.

Our study demonstrates several strengths. Firstly, our cohort analysis draws from ten NHANES cycles spanning 1999 to 2018, ensuring robustness with ample data and a lengthy investigative period. Secondly, our choice of the HALP score as a parameter offers a comprehensive assessment of both inflammation and nutritional status, surpassing single inflammation metrics. This approach enriches the evaluation of cancer survivors’ physical well-being and enhances outcome prediction reliability. Thirdly, our study pioneers the comprehensive examination of HALP scores’ association with all-cause and specific mortalities in cancer survivors, including cancer, cardio-cerebrovascular disease, and respiratory disease mortalities. These findings provide invaluable insights for prognostic management in this population. Lastly, employing diverse analytical methodologies such as RCS nonlinear analysis, subgroup scrutiny, and Kaplan-Meier analysis further consolidates the HALP score’s validity as a reliable mortality risk indicator for cancer survivors.

Several limitations must be acknowledged in our study. Firstly, NHANES data relies on self-reports from patients, which could introduce recall bias. Secondly, despite our efforts to adjust for known confounding factors such as age, gender, and smoking status, there may still be unidentified confounders affecting our results. Thirdly, the HALP score utilized in our study was derived from a single complete blood count parameter and serum albumin measurement, which may not fully capture individuals’ overall health status, potentially leading to bias.

## 5 Conclusions

By conducting a comprehensive survey of cancer survivors across the United States, our study unveiled a significant nonlinear relationship between HALP scores and both all-cause and cause-specific mortality. Elevated HALP scores were consistently associated with lower long-term mortality rates among cancer survivors. Therefore, the HALP score emerges as a practical and cost-effective tool for identifying high-risk groups within this population. Our findings underscore the promising potential of HALP scores in prognosticating outcomes for cancer survivors, offering valuable insights for clinical decision-making in this demographic.

## Data Availability

The original contributions presented in the study are included in the article/supplementary material. Further inquiries can be directed to the corresponding authors.
